# Regional diversity of complex dissolved organic matter across forested hemiboreal headwater streams

**DOI:** 10.1038/s41598-018-34272-3

**Published:** 2018-10-30

**Authors:** Jeffrey A. Hawkes, Nikola Radoman, Jonas Bergquist, Marcus B. Wallin, Lars J. Tranvik, Stefan Löfgren

**Affiliations:** 1 Analytical Chemistry, Department of Chemistry - BMC, Uppsala University, Uppsala, Sweden; 2 Department of Earth Sciences, Uppsala University, Uppsala, Sweden; 3 Limnology, Department of Ecology and Genetics, Uppsala University, Uppsala, Sweden; 4 Department of Aquatic Sciences and Assessment; Section for Geochemistry and Hydrology, Swedish University of Agricultural Sciences (SLU), Uppsala, Sweden; 5 Present Address: Department of Environmental Science and Analytical Chemistry, Stockholm University, Stockholm, Sweden

## Abstract

Dissolved organic matter (DOM) from soils enters the aquatic environment via headwater streams. Thereafter, it is gradually transformed, removed by sedimentation, and mineralised. Due to the proximity to the terrestrial source and short water residence time, the extent of transformation is minimal in headwaters. DOM has variable composition across inland waters, but the amount of variability in the terrestrial end member is unknown. This gap in knowledge is crucial considering the potential impact large variability would have on modelling DOM degradation. Here, we used a novel liquid chromatography –mass spectrometry method to characterise DOM in 74 randomly selected, forested headwater streams in an 87,000 km^2^ region of southeast Sweden. We found a large degree of sample similarity across this region, with Bray-Curtis dissimilarity values averaging 8.4 ± 3.0% (mean ± SD). The identified variability could be reduced to two principle coordinates, correlating to varying groundwater flow-paths and regional mean temperature. Our results indicate that despite reproducible effects of groundwater geochemistry and climate, the composition of DOM is remarkably similar across catchments already as it leaves the terrestrial environment, rather than becoming homogeneous as different headwaters and sub-catchments mix.

## Introduction

Inland waters are increasingly recognised as an important component of the global carbon cycle, and it is estimated that riverine flux of organic carbon to the sea represents only a fraction (<20%) of the 5.1 Pg yr^−1^ that is delivered from the terrestrial environment. The bulk is mineralised to CO_2_ or buried in riverine and lacustrine sediments^1–5^. The mineralization and sedimentation of terrestrial dissolved organic carbon (tDOC) are intrinsically controlled due to the structure of the organic molecules^6,7^ and extrinsically mediated by the environment^8^, leading to complex bulk decay rates spanning several orders of magnitude in inland waters^9^. These processes are spatially and temporally variable, and the source^10–13^ and reactivity^14–16^ of this carbon resource are among the most extensively researched topics in contemporary biogeochemistry.

Headwater streams are a fundamental part of river networks, being the point at which groundwater leaves the soils and turns into surface water to form the earliest stages of the river. The flow rate and geochemistry of headwater streams are heavily affected by groundwater table depth^17–19^, land use and climate^20^, and their composition forms the basis of the entire river network. In Sweden, first and second order streams make up 78% of the total length of all rivers^5^. In organic carbon terms, headwater streams are absolutely dominated by terrestrial dissolved organic matter (tDOM) that has been previously altered in soils and groundwater^12,13,21^. Primary production and other in-stream processes are highly constrained by the short water residence time in low order streams^22^, and only begin to significantly affect organic matter in streams at higher order stream level^23,24^. This makes headwater streams ideal sites to examine the geochemical controls on the original character of complex tDOM being transported to aquatic systems before further complication due to *in-situ* production and transformation of the organic matter that occurs in downstream rivers and lakes^25^.

The composition and concentration of tDOM in headwater streams depends on soil chemistry and groundwater flow paths yielding different contribution of deep and shallow groundwater inputs^26^. Changes in soil pore water ionic strength and air temperature over the last decades have led to an increased flux of tDOM from soils to inland waters^27,28^, in many cases exceeding 0.15 mg L^−1^ yr^−1^ (ref.^29^). Transport through deep groundwater aquifers tends to remove tDOM due to interaction with mineral cations (Al^3+^, Fe^3+^, Ca^2+^)^30,31^, and it has been demonstrated that aromatic and carboxylic functional groups in the complex organic mixture have a higher tendency to sorb onto mineral and soil surfaces^32–34^.

While experimental studies have demonstrated the chemical fractionation of tDOM under various controlled conditions, few studies have analysed the molecular composition of headwater stream tDOM^21,35,36^, and no studies have sampled a sufficiently large number of headwater streams in one region to determine potentially subtle effects of geochemistry on the resulting complex mixture.

Compositional analysis of DOM can be approached using high resolution mass spectrometry to determine signal intensities of thousands of molecular formulas in each aquatic sample^8,21,37^. This is usually achieved after solid phase extraction of material and analysis via Electrospray Ionisation -Fourier Transform – Ion Cyclotron Resonance - Mass Spectrometry (ESI-FT-ICR-MS). We have recently evaluated the use of the Orbitrap Mass Spectrometer for the same task^38^ and coupled online chromatographic fractionation of the material without need for prior solid phase extraction^39^. This allows robust and reproducible analysis of large numbers of filtered freshwater samples either without modification or after pre-concentration by vacuum centrifuge. The polarity fractionation of the samples allows a deeper investigation of the isomeric complexity of the sample, but does not resolve individual isomers^40^, and still requires statistical analysis of compositional data, rather than leading to a complete understanding of DOM molecular concentrations.

In this study we measured the relative abundance of thousands of molecular formulas over three polarity fractions in 74 randomly selected forested headwater streams in southeast Sweden using online high performance liquid chromatography- high resolution mass spectrometry (HPLC-HRMS)^39^. The HPLC-HRMS data were evaluated along with a range of geochemical, geographical and meteorological characteristics in order to determine how hydrology and geochemistry control the concentration and character of tDOM in headwater streams, and therefore the entire river network.

## Results and Discussion

### Sample catchment characteristics and geochemistry

**Figure 1:**
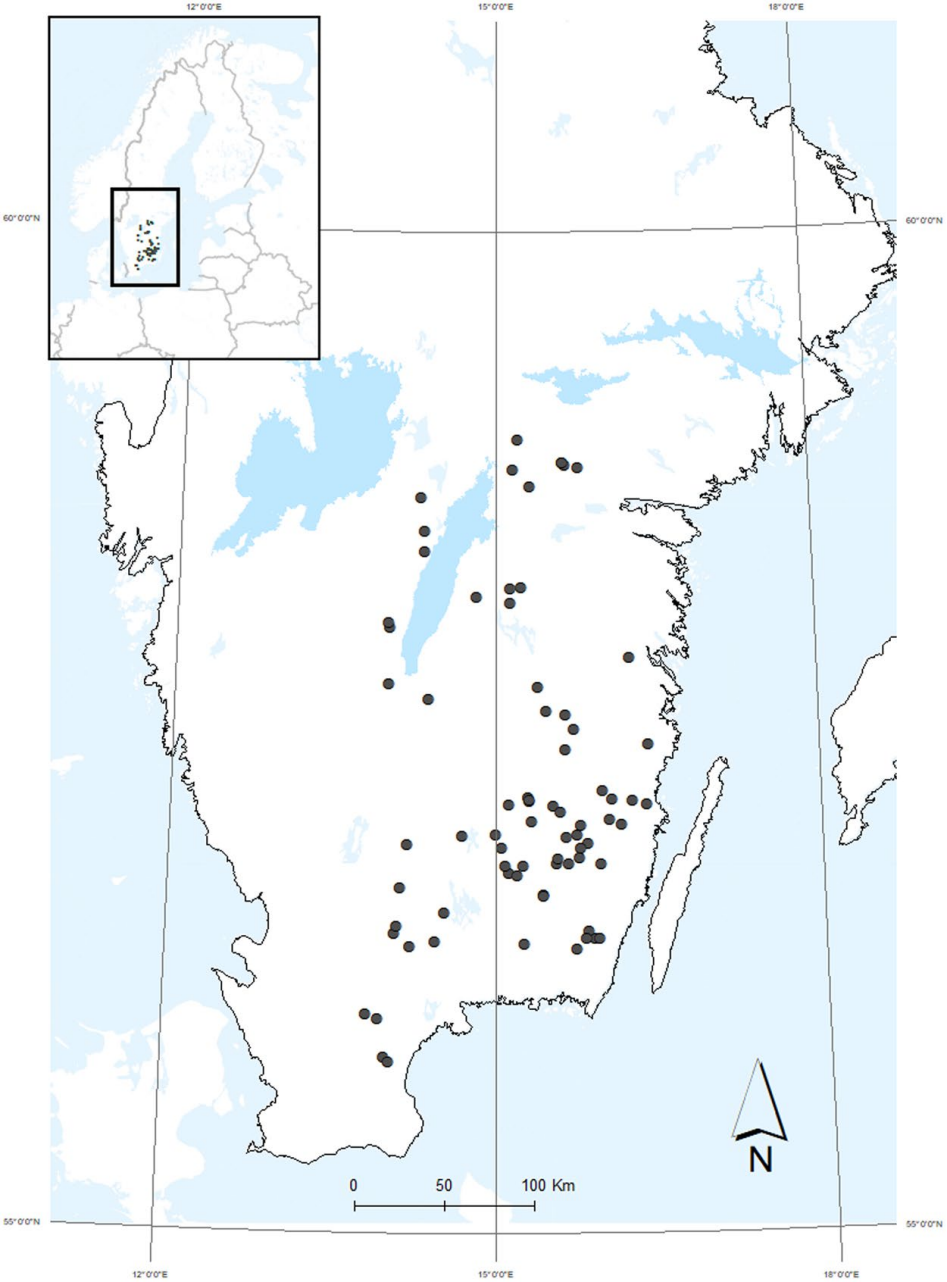
Map of sampling locations in southeast Sweden.

The headwater streams were sampled over an 87,000 km^2^ region (Fig. 1) using a random selection technique, resulting in a wide range of catchment characteristics and geochemistry (Table 1). The catchments were dominated by forests, with only 25% of the headwater catchments having more than 2% wetland area. Due to the selection criteria, surface water, agriculture and urban areas accounted for negligible area. The catchments were primarily located on slowly weathering soils (Podzol, Histosol, Leptosol, Arenosol, and Regosol) and bedrock (granite and gneiss), typical for forested areas in southern Sweden, and with limited influence of lime or other calcium bearing soil types^41^. Based on long-term data for the period 1961–1990, annual mean temperature and precipitation varies in the range 5–7 °C and 600–700 mm (ref.^42^). The evapotranspiration is in the range 400–500 mm yielding a low run off of 100–200 mm. Hence this region, especially along the coast to the Baltic Sea, is the driest of Sweden. The vegetation period (average number of days with an air temperature >5 °C) varies between 180–210 days and 15–25% of the precipitation fall in the form of snow^42^. The dominant tree species in Southeast Sweden are Norway spruce (*Picea abies* (L.) Karst.), Scots pine (*Pinus- sylvestris* L.) and birch (*Betula spp*.). Except for a few years after clear-cutting, these tree species dominate the biomass and production in Swedish forests. Note that the assessment of wetland coverage should be considered as a minimum estimate, and does not consider peat soils in forested areas, as these are counted as forest area by the satellite imaging technique.

**Table 1:** Catchment characteristics and headwater stream geochemistry.

	Range	Median
Catchment area (km^2^)	0.95–7.25	2.34
% Forested area	48–99	83
% Clear-cut area	1–34	14
% Wetland area	0–44	0
TOC (mg/L)	6.5–63.3	27.9
Total Ca (meq/L)	0.09–3.10	0.25
Total Fe (mg/L)	0.04–3.8	0.8
pH	4.01–7.40	4.97
ΣBC (meq/L)	0.29–3.84	0.67
TOC:ΣBC+ (mmol:meq/L)	0.3–12.7	3.8

ΣBC = sum of base cations (Ca^2+^, Mg^2+^, K^+^, Na^+^).

The TOC concentration ranged between 6.5–63.3 mg/L in a distribution skewed towards the lower end. Run-off influenced by deep groundwater flow-paths led to higher base cation (Ca^2+^, Mg^2+^, K^+^, Na^+^  = ΣBC) concentrations in some samples^13^, leading to a highly skewed distribution with median 0.67 meq/l ΣBC. Titrated alkalinity (pH_ref._ = 5.6) was low, often negative, leading to low sample pH with more than half of the streams having pH < 5.

### Liquid Chromatography – mass spectrometry separation and analysis of samples

**Figure 2:**
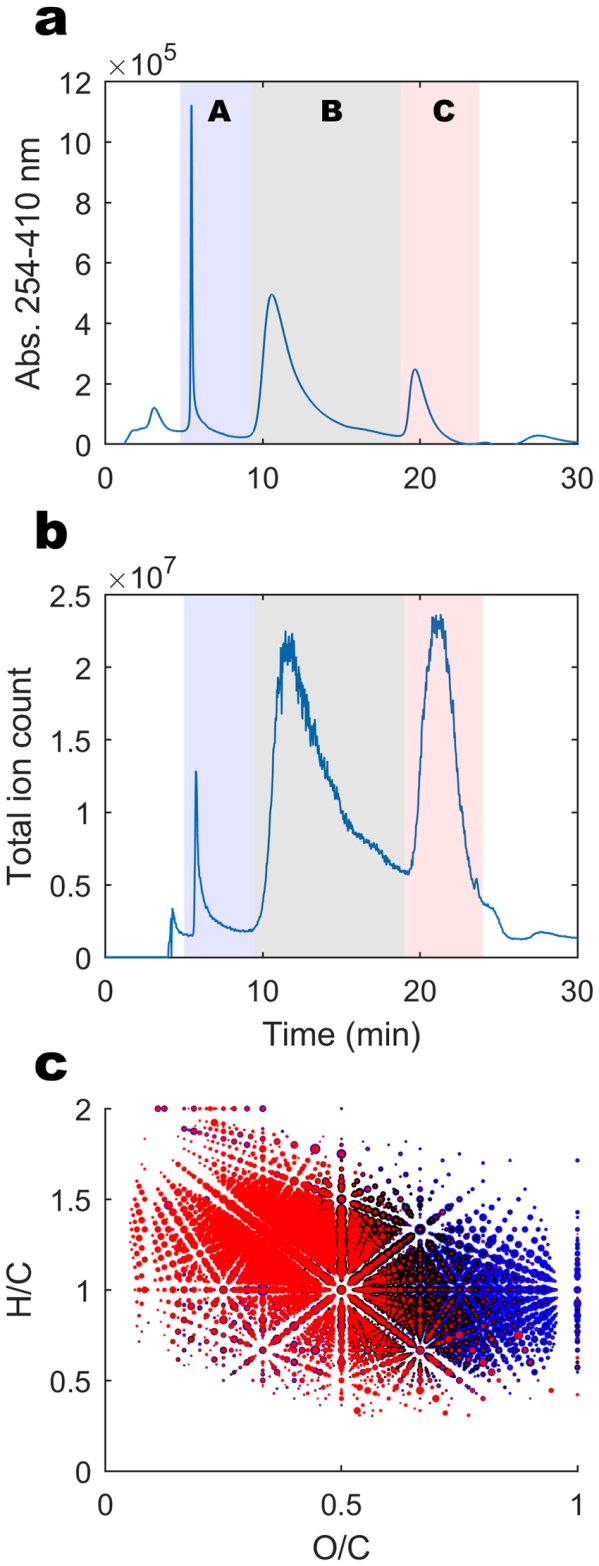
DOM characteristics of one exemplary sample (SO_023) in the three polarity fractions (Fraction A: blue, Fraction B: black, Fraction C: red). (**a**) Absorbance at 254 nm of organic matter eluting from the column over time. (**b**) Total ion count by mass spectrometry of the same material, with polarity fractions divided *in-silico* indicated by shaded areas. (**c**) Van Krevelen diagram showing H/C vs O/C atomic ratios of all compounds detected in the three fractions, with colours indicating the fraction and point size showing log(relative intensity).

Sample DOM was characterised by high resolution mass spectrometry after elution from a reversed phase column in three main fractions (Fig. 2): Poorly retained, hydrophilic material (Fraction A), material that was retained but eluted in >20% acetonitrile (Fraction B) and the most hydrophobic material that was retained and eluted only when acetonitrile was increased above 45% (Fraction C). Note that hydrophobicity is defined here conditional on the pH of sample loading (3.35), not the pH of the stream. The average light absorbance of the material decreased with hydrophobicity due the loss of carboxyl and unsaturated (π electron) functionality (Fig. 2).

The polarity fractionation of the sample prior to mass spectrometry analysis allows a greater number of formulas to be assigned, particularly in the more hydrophobic fractions^39^. It also improves the detection limit by decreasing the molecular complexity at the ESI source and in each transient analysis in the Orbitrap, where there is a maximum of 1 × 10^6^ ions analysed. The resulting data is a matrix of intensities with the number of rows set by the number of formulas considered and number of columns set by the chromatographic fractions (in this case, 6220 × 3 = 18660 variables). The increased number of variables leads to deeper sensitivity to sample differences in multivariate statistical analysis.

The intensity data was normalised so that all assigned masses across the three fractions summed to a unitless value of 1 × 10^6^. The data in Fig. 2 was taken from an average sample with the lowest average Bray-Curtis dissimilarity of DOM to all other samples, designated sample SO_023. This sample had 24.8 mg/L TOC and 0.25 meq/L Ca^2+^. In this average sample, fraction A, B and C contributed 0.5, 6.0 and 3.5 × 10^5^ units of signal, respectively, but these values do not correspond to the actual concentration differences of these polarity fractions, which would have to be measured by more quantitative means after fraction collection. The scale of Fraction A was largely controlled by the pH of mobile phase A; with greater proton activity leading to better neutralization and retention of organic acids. This indicates that the pKa of the fraction A material is lower than 4 and is largely constituted by conjugated carboxylic acids^43^. This is consistent with the high O/C ratio, low hydrogen saturation (Table 2) and previous studies that demonstrate the importance of carboxylic functionality in DOM by fragmentation and methylation reactions^40,43^. In relation to stream water acidity (pH > 4.0), fraction A is a relatively strong acid having the potential to decrease pH in all streams where the buffer capacity remains low^44^.

**Table 2:** Average sample data for three hydrophobicity fractions in sample SO_023.

	Fraction A	Fraction B	Fraction C
*m/z*_wa_	365 (281–423)	367 (297–421)	381 (307–441)
O/C_wa_	0.70 (0.58–0.79)	0.50 (0.41–0.58)	0.38 (0.32–0.43)
H/C_wa_	1.00 (0.83–1.13)	1.20 (1.00–1.33)	1.29 (1.14–1.38)

Subscript ‘wa’ signifies the values are weight-averaged based on peak intensities. Displayed are weighted average median with interquartile range within brackets.

The increasingly hydrophobic material was on average higher mass, more saturated and had lower oxygen functionality (Table 2), typical of DOM from higher pH soils^45^. All of the material recovered can be considered as rather unsaturated in comparison to most biologically labile organic matter, which generally has an H/C ratio higher than 1.5^46^. The DOM was composed of unsaturated compounds (H/C 1.0–1.5), generally in fractions B-C, and highly unsaturated, carboxyl rich compounds (H/C < 1.0, O/C > 0.5) in fraction A. The more saturated compounds (H/C > 1.0) tend to be recalcitrant and persist in freshwater and marine environments^7^. The highly unsaturated, carboxyl rich compounds reflect the origin of tDOM in terrestrial plants, which tend to release highly phenolic material to the watershed^8^ that is subsequently oxidised and carboxylated in soils^47,48^. There was significant overlap in terms of presence and absence (not intensity) in molecular formulas between the three fractions, due to the extreme isomeric complexity of the molecular mixture^39,40,49^. Structural isomers for individual formulas were spread between the three fractions. We have previously confirmed that specific (purchased) compounds that are spiked into the complex mixture are indeed retained in reproducible and narrow elution times^39,40^. Of the 4305 formulas detected in this sample, 2314 were present in all three fractions, but usually at highly differing intensities (Fig. 2c). Only 21, 10 and 253 formulas were unique to the three fractions A, B and C, respectively. This overlap is due to the incredible complexity of the mixture and the structural diversity hidden behind each formula, and not primarily due to shortcomings in the separation.

### Regional sample variability

Despite the large range in sample TOC concentrations, inter-sample variability (after TOC normalization by dilution and normalization of the data) was low, with an interquartile range of dissimilarities of 6.2–10.0% (Fig. SI2) and no values passing 23.3%. For context, Bray Curtis dissimilarity between lakes with water residence time less than *vs*. more than 1 year is greater than 30%^50^. Also, terrestrial and marine DOM have been found to differ >60%^38^. Both of these values were generated from direct infusion data, so strict comparison is not possible with data from HPLC-HRMS.

Over the three polarity fractions, 1751, 2851 and 2382 molecular masses (in total 6984 fraction specific masses) were present in all 74 samples. While this only represents 39% of fraction specific masses identified in the dataset in at least one sample, these 6984 peaks constituted essentially all of the ionizable organic matter by making up 99.2 ± 0.2% (mean ± SD) of total intensity in the samples. Hence, most of the difference between streams in presence or absence of peaks could be attributed to rare, low abundance peaks. Together, these metrics suggest that the quality of DOM across this large geographical region was highly similar, albeit at very different concentrations. Classical multidimensional scaling of the Bray Curtis dissimilarity matrix revealed that just three principle coordinates could describe 76% of the data variability between the 74 samples. These three coordinates described 48, 20 and 7% of the data variability respectively, with subsequent coordinates describing less than 5% of the data each and presumably accounting only for analytical variability (Fig. SI2).

**Figure 3:**
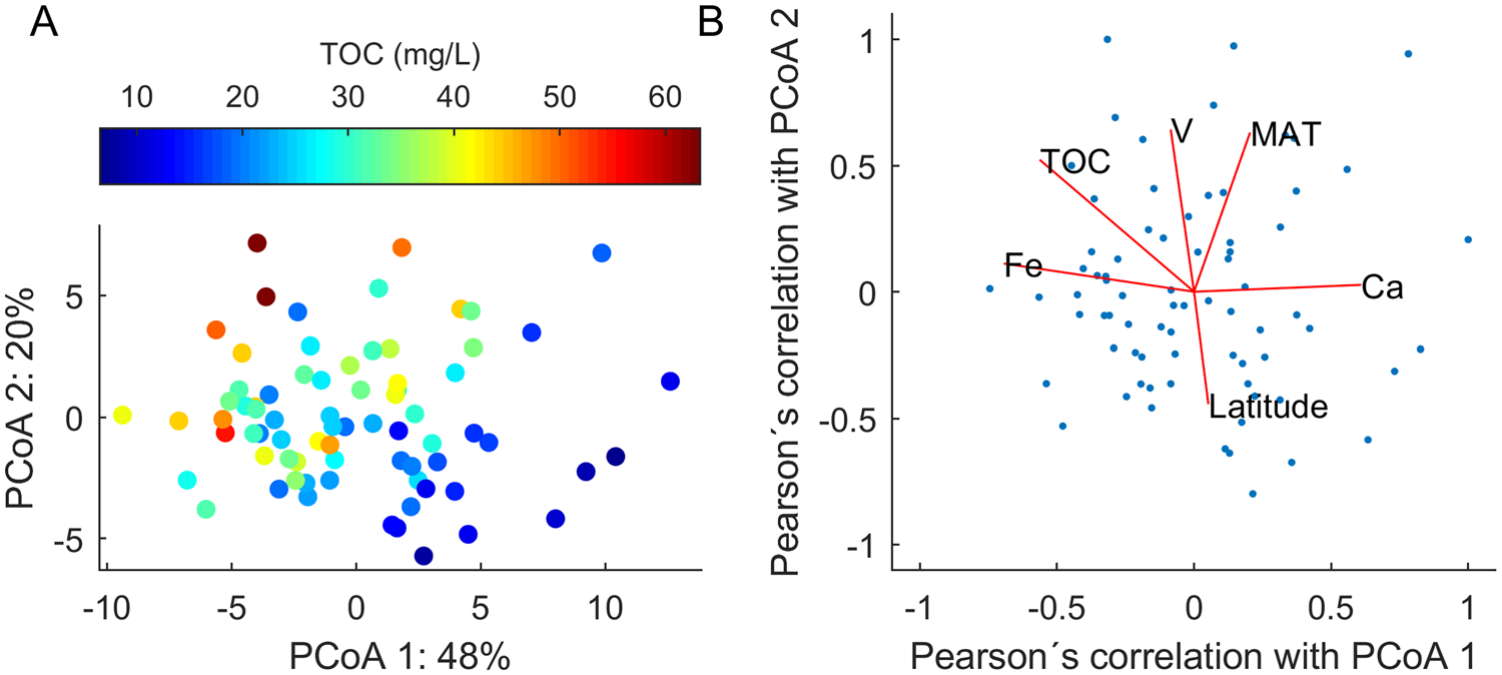
(**A**) Principle coordinate diagram showing the 74 sample positions on the first two coordinates, which account for 68% of all DOM compositional variability in the dataset. Sample TOC concentration is displayed in colour. (**B**) Pearson’s correlation values of selected environmental/geochemical parameters with the first two principle coordinates, indicated by lines that lead from the centre to the x,y values of Rho. The normalised scores of the samples in the same two dimensions are overlaid in small blue dots. Correlation values for all parameters can be found in the supplementary information.

**Figure 4:**
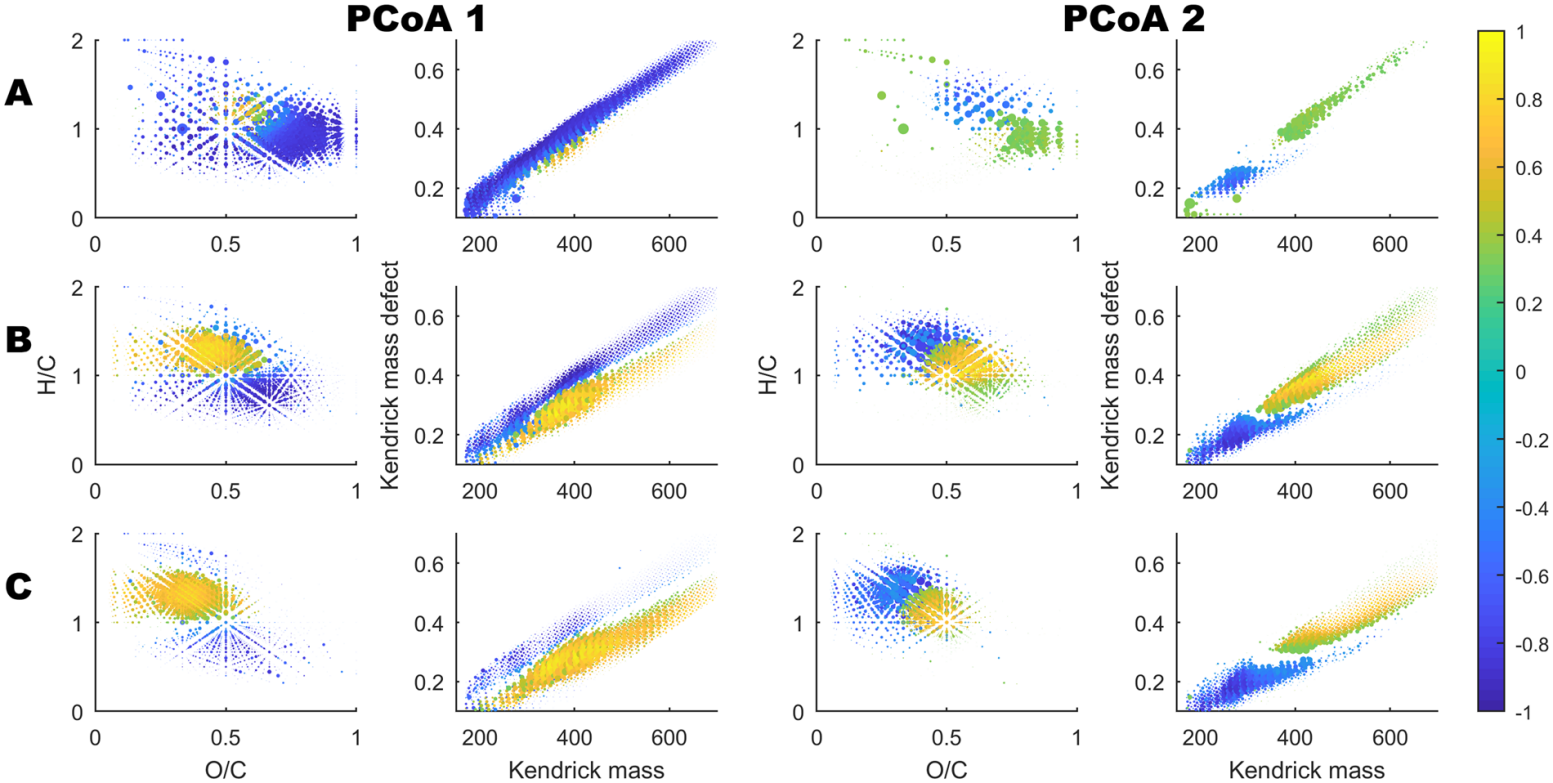
Van Krevelen and Kendrick diagrams of molecular formulas in each of the three polarity fractions (**A**–**C**). Points are shown for molecular formulas whose intensities correlated significantly (p < 0.001) with either PCoA 1 (left) or PCoA 2 (right). The size of the point is proportional to the log(normalised intensity) and the colour of the point indicates the Pearson’s correlation rho (legend at right). PCoA 1 correlates negatively with oxygen saturation and positively with hydrogen saturation, whereas PCoA 2 correlates with molecular mass.

The first principle coordinate (PCoA 1) correlated with the concentration of major ions including Ca^2+^, SO_4_^2−^ and Mg^+^, and also pH, alkalinity, and conductivity, and negatively correlated with Fe and Pb. PCoA2 was positively correlated with mean annual air temperature (MAT) at the sampling site, V, Al, Se and Cr, and negatively correlated with (northerly) latitude (Fig. 3). The correlations mentioned had a *p*-value < 0.001 over the 74 samples (Pearson’s Rho > ± 0.375). All correlation data between the PCoAs and environmental factors are reported in the supplementary information. Individual molecular formulas within each of the three polarity fractions could also be correlated with the principal coordinates (or the environmental factors), revealing important trends in DOM quality according to these geochemical gradients (Fig. 4).

In this quality context, the first principle coordinate had a clear positive correlation with hydrogen saturation in polarity fractions B–C, and was negatively correlated with the most oxygen rich (carboxyl rich) compounds (O/C > 0.5) that are commonly found in fractions A–B. The proportion of total ion count found in fraction A decreased substantially along PCoA1, making up ~6.3% of total intensity in the higher TOC samples, and just 3.0% in the higher ionic strength samples (SI Fig. 3). In geochemical context, this means that the samples that were rich in ionic strength/alkalinity were also richer in saturated and oxygen poor compounds - particularly those with H/C > 1.0 and O/C < 0.5, in fractions B–C. The opposite can be stated for samples rich in Fe, Pb and TOC. The second principle coordinate had a clear positive correlation with mass, with a large shift in relative intensities occurring around *m/z* 350 and Kendrick Mass Defect 0.3. The more southern samples, from slightly warmer climates, were richer in high mass compounds and also V, Al, Se and Cr. Most of these second coordinate quality changes affected the more saturated compounds with H/C > 1.0 (Fig. 4).

Polar, oxygen rich, unsaturated molecules were thus less abundant in samples with relatively high ionic strength. Ionic strength in headwater streams is typically sourced from sub-surface mineral soils, indicating that deeper groundwater flow paths result in sorption and ligand binding of compounds with high carboxyl functionality to soil particle bound metals^51^. The abundance of calcium bearing soils is minimal in this study region, meaning there is a low probability that our results are confounded by surface sources of calcium.

Counter-intuitively, the more hydrophobic, saturated constituents remained dissolved in the groundwater, and were proportionally of higher abundance in headwater streams with a greater deep groundwater contribution. This removal mechanism preferentially removes the same carboxyl and aromatic functionality as photo-degradation, and leads to changes that resemble those found in samples that have been processed in surface waters^7,12,46,52^. With respect to surface water runoff generation, the timescale of groundwater residence time is generally confined to <1 yr in this region^26^, whereas water residence times in lakes can reach tens of years^9^. The extent of change found in our samples is fairly low, but at this stage we cannot assess whether this is limited by residence time or geology^46^.

Conversely, high Fe and Pb samples were associated with low values on PCoA1 and therefore more oxygen rich components. It stands to reason that high carboxyl functionality is associated with heavy metal transport, but usually high Fe is an indication of reducing conditions^53^, probably reflecting lateral influx of shallow groundwater from organic rich riparian zones^17^. The presence of highly oxidised DOM in reduced waters suggests that the formation of the carboxylic rich DOM occurs in the hydraulically unsaturated zone, possibly in the B-horizon of oxidised forest soils^54^ early on in the diagenesis of deposited terrestrial organic matter^47,48^.

These results support previous evidence that the bulk of dissolved organic matter in headwater streams in forested landscapes is previously formed in soils and is thereafter quite stable^17,22^. Our findings of slightly different DOM composition between streams with different relative influence of deeper groundwater flow paths, as indicated by ionic composition, imply that DOM that has been ‘pre-aged’ through deep groundwaters this way will attenuate less light, have lower carboxyl functionality and therefore lower heavy metal binding/carrying capacity and will be less photodegradable and surface active. Catchments with surficial groundwater flow-paths through organic rich soils will export relatively browner waters with less altered organic matter leading to higher heavy metal transport capacity per mole of carbon. It is also important to note that the most extensive differences in DOM observed in this study still left the DOM character highly similar among samples. When analysed in direct infusion mode after simply diluting the sample in acetonitrile (50%) and ammonia (0.1%), ‘tannin-like’ molecules with H/C < 0.8, O/C > 0.75 were not detected in the low TOC sample (Fig. SI4), whereas they were detected by the HPLC-HRMS method employed here. The greater sensitivity over the whole polarity range provided by the LC-MS method may decrease the importance of ion suppression^39^, making samples more comparable, at least in terms of presence and absence of peaks^35^. This may also have led to some extent to the higher observed similarity of samples, suggesting that the beta diversity (inter-sample diversity) of DOM may be overestimated by direct infusion HRMS. The apparent similarity may also be exaggerated (by both LC-HRMS and direct infusion approaches) due to the effect of isomeric averaging of the thousands of structures that make up each peak, hiding more varied behaviour within each molecular mass^40,55^.

PCoA 2, which was driven largely by latitude and long-term mean annual temperature (MAT), correlated well (ρ 0.62, *p* < 1 × 10^−8^) with an important fraction of higher molecular mass (> 350 Da) material, generally with hydrogen saturation >1.0. The MAT variability between the samples was fairly low, between 5–7 °C, and the southern sites are consistently ~2 °C warmer than the northernmost sites. MAT was not correlated with DOC concentrations, confirming previous observations across Sweden^56^, but may have an impact on DOM quality due to differences in adsorption or enzymatic activity in soils^57–60^. Typically, increased microbial respiration of DOM would be expected to decrease molecular mass^61^, although some evidence suggests that high molecular weight material is preserved/low molecular weight material is removed in headwater streams from forest soils under incubation conditions^62^. In addition to possible explanations related to soil chemistry and *in-situ* microbiology, it should be noted that MAT positively co-varies with vegetation period length and forest and litter production levels^63^, resulting in a difference of ~4 m^3^ ha^−1^ yr^−1^ in mean site quality (SI) from the most southern to most northern catchment (Swedish Statistical Yearbook of Forestry, 2014), reflecting a much larger potential for OM production (and decomposition) by the trees in the south (SI = ~11 m^3^ ha^−1^ yr^−1^) compared with in the north (SI = ~7 m^3^ ha^−1^ yr^−1^). We speculate that this difference has the potential to increase the overall influx of higher molecular mass DOM to the soils. This aspect of our findings adds some evidence to recently observed temperature effects on DOM quality in soils and rivers^45,64^, but still requires considerable further investigation. If this relationship is causal and reproducible over such small temperature ranges then changes to temperatures (and therefore litter production levels) that are expected from near-future climate change could have a noticeable impact on the character, and especially the mass profile of riverine DOM.

**Figure 5:**
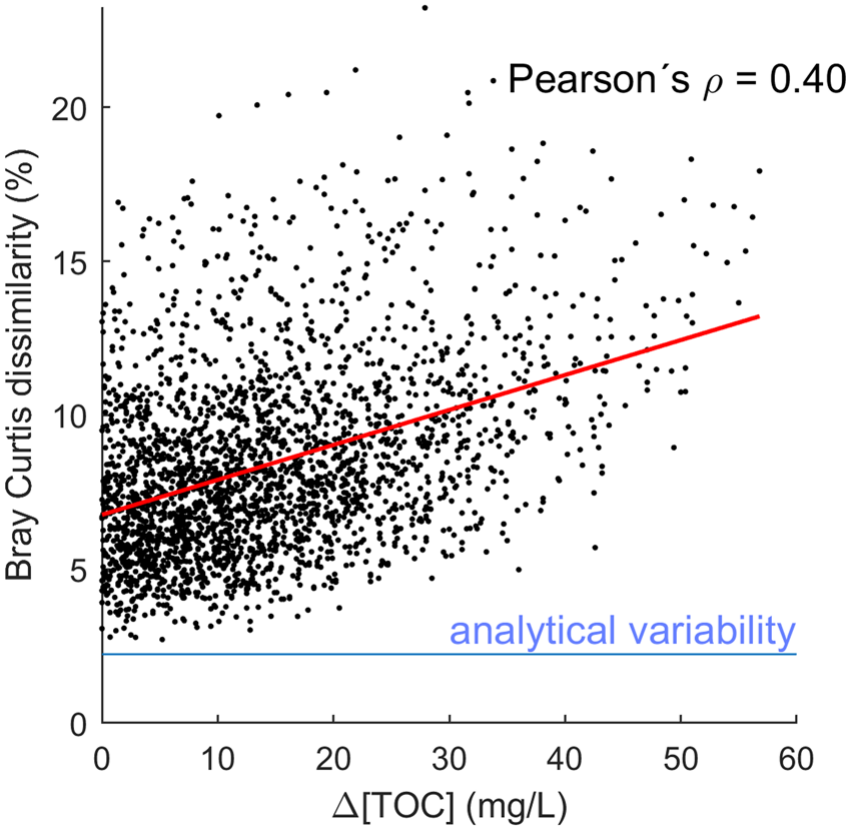
Pairwise Bray-Curtis dissimilarity vs. Pairwise difference in TOC. TOC difference is a poor predictor of Bray Curtis dissimilarity.

Notwithstanding these subtle differences, that were reproducible across the 87,000 km^2^ region, all samples in this study were rather similar. This similarity was found despite an order of magnitude range in TOC concentrations (~7–70 mg/L), with the ubiquitous molecular masses making up an average of 99% of intensity and Bray-Curtis dissimilarity not passing 23.3%, even incorporating data separated over three polarity/acidity fractions. We assume that the large range in TOC concentration is caused by variability in peat soil abundance, which could not be measured in this study. The concentration difference of TOC is likely to have various geochemical effects on the aqueous environment, including acidity, light retention and trace metal solubility, but the rate constant of its chemical or biological removal is unlikely to be different due to its high apparent compositional similarity. The % wetland parameter is likely to be a low estimate due to the nature of satellite imaging (peat soils in forests are counted only as forests). Overall, TOC was not well predicted by any unrelated variables, in particular land use parameters. TOC in turn was a poor predictor of Bray Curtis dissimilarity of sample DOM composition (Fig. 5).

The overall regional similarity suggests that headwater or terrestrial DOM in this kind of forested hemiboreal environment is similar enough to be used as a tracer for the headwater end member of DOM in comparative studies within and among forested catchments, and in degradation studies. Here, we demonstrate the similarity across a wide selection of forested catchments draining to hemiboreal streams. Possibly the similarity of tDOM extends to other boreal environments, and a comparison study of a large number of streams from different biomes is an important next step^21^. Headwater tDOM is likely masked by urban and agricultural runoff downstream, making the study of its degradation and transport challenging. Our results demonstrate that the tDOM mixture exported to headwaters streams, although highly complex, exhibits substantial similarity across the forested landscape. In ecological terms, this corresponds to high alpha diversity (within each sample) and low beta diversity (across forested regions). Accordingly, we expect the DOM of different forested headwaters to react in similar ways during microbial and chemical degradation^6^, and we posit that the composition of DOM should be expected to be rather similar across forested catchments already as it leaves the terrestrial environment, rather than becoming homogenous as different headwaters and sub-catchments mix^52^. This is a crucial finding for modelling studies that assess the likelihood that terrestrial organic carbon from forested regions is respired or sedimented in streams, as our results suggest that they can now assume homogeneity of the terrestrial source material.

## Methods

### Sample collection and geochemical data

Samples were collected late in the autumn 2016 (November 17- December 1) from the surface (0–0.3 m, depth) of 74 forested headwater streams across the southeast of Sweden (Fig. 1), as a part of a research study coordinated and conducted by the Swedish University of Agricultural Sciences (SLU). 100 candidate streams for sample collection were selected randomly using a national virtual database of stream networks, VIVIAN^65^. The initial selection criteria were that streams must be longer than 2500 m, from catchment areas with water surface area ≤1%, agricultural land ≤5% and without urban influence. For a detailed description of the selection methodology see the supplementary information and Löfgren *et al*. (2014).

Geochemical analysis of samples (pH, conductivity, turbidity, TOC, inorganic ions, metals, and absorbance) was performed based on accredited methods by the Department of Aquatic Sciences and Assessment, SLU (https://www.slu.se/en/departments/aquatic-sciences-assessment/laboratories/geochemical-laboratory/water-chemical-analyses/). Arsenic, cobalt, cadmium, chromium, copper, nickel, lead, selenium, zinc, vanadium and uranium were determined by ICP-MS. Aluminium, iron, manganese, calcium, magnesium, potassium, sodium and silicon were determined by ICP-AES. Sulfate, chloride and fluoride were determined by ion chromatography. Total organic carbon and total nitrogen were determined by combustion methods using a Shimadzu TOC/TN analyser.

### Catchment characteristics

Average climate data for the period 1961–1990 were used and obtained from the Swedish Meteorological and Hydrological Institute (SMHI). Land cover distribution within each catchment was based on the digital version of the Swedish land cover map produced by Metria (GSD-Marktäckedata; http://mdp.vic-metria.nu/miljodataportalen/) classified from satellite images (25 × 25 m pixel). Data on tree biomass and annually clear-cut area were made available as digital maps by the Swedish Forest Agency (SFA, https://www.skogsstyrelsen.se/sjalvservice/karttjanster/skogliga-grunddata/). Tree biomass is modelled based on data from LIDAR scanned digital elevation maps (2 × 2 m pixel) and ground truth data from the Swedish National Forest Inventory (NFI). Clear-cut area is classified as the accumulated final felled area during the period 2007–2016.

### Sample preparation

Solutions were prepared using reagent grade chemicals and ultra-pure water (>18 MΩ cm^−1^). Samples were collected in November 2016, were stored unfiltered in the dark at 4 °C and were prepared and analysed on the 23^rd^–26^th^ April 2017. The samples were measured into 2 ml Eppendorf vials so that 11.25 µg organic carbon was present (e.g. 1.125 ml sample if TOC = 10 mg/l). Two blanks of 2 ml ultrapure water (MilliQ, Millipore) were taken to determine contamination peaks. Samples and blanks were evaporated to dryness in a vacuum centrifuge (Eppendorf Concentrator Plus) at 45 °C and reconstituted in 150 µL MilliQ water with 1% (v/v) formic acid, so that resulting concentration for all samples was 75 mg/L of TOC. This was done in order to inject the same amount of TOC into LC-MS system for each sample. Some flocculation of organic carbon was apparent in the more TOC rich samples, and possibly the most hydrophobic moieties of the DOM flocculated selectively. We did not quantify this effect, but assume that it is a natural process that would also affect organic carbon in the natural environment, leading to loss by sedimentation. This study only focuses on the DOM which remained dissolved, and flocs were avoided when taking samples for drying. The main effect this would have on our data is due to the dilution correction applied to the samples, which would lead to these samples having a lower analytical abundance of DOM than expected. Normalization of the data will have accounted for this effect.

### Reversed-Phase High Performance Liquid Chromatography (RP-HPLC) with Diode Array Detection (DAD)

Chromatographic separation of the samples was carried out on an Agilent 1100 Series system with a polymeric polystyrene/divinylbenzene reversed phase column (150 × 1.0) mm, 3 µm bed size, 100 Å pore size (Agilent, PLRP-S). Samples were analysed in random order with blanks and three analyses of Suwannee River Fulvic Acid (SRFA) reference material (2 × 75 mg/L C, 1 × 150 mg/L C) placed randomly between samples. Samples (80 µL) were loaded at 100 µL/min and were eluted in a stepped gradient at 40 µL/min formed by two mobile phases leading to the elution of three fractions of material (broadly, 0% B, 20% B, >45% B). This is a high loading volume compared with the flow rate, but we have previously confirmed that the retention factor of DOM is high enough for this not to affect peak shape^39,40^. Mobile phase A was 0.1% (v/v) formic acid, 0.05% (v/v) ammonia, 5% (v/v) of acetonitrile, with resulting pH = 3.35. Mobile phase B was acetonitrile. The elution profile is described in detail in the supplementary information. UV-visible absorption spectra were measured with Agilent 1100 diode array detector at 254 nm with 410 nm as the reference absorption. The total analysis time per sample was 34 minutes, and 80 samples, blanks and reference samples were analyzed in 48 hours. We have noticed in other sample sets that some column and ESI cleaning is required for more alkaline samples, but for headwater streams no signal suppression was observed.

### FT-Orbitrap-MS analysis

MS analysis was conducted following the chromatographic elution with an Orbitrap LTQ-Velos-Pro (Thermo Scientific, Germany). Ions were formed by electrospray ionization in negative mode at a spray voltage of −3.1 kV. Method setup and data acquisition were performed using Thermo Fisher Scientific Xcalibur^TM^ software (version 3.0). External calibration of the instrument’s mass/charge (*m/z*) axis was performed at the start of the analysis using the manufacturer’s calibration mixture for negative mode. Mass spectra were collected after the initial 4 minutes of eluent was diverted to waste. Transients were collected over the following 30 minutes for each LC-MS run, with a range of 150–1000 *m/z* and instrumental resolution setting of 100 000. Sample *m/z* values were recalibrated using ReCal Offline software (Thermo Fisher) using six masses from 251–493 that were found in all samples.

Sample data were processed in MATLAB (Version 2017b). Thermo Fisher Raw files were converted to mzXML files with ReAdW, and were imported into MATLAB with the mzxmlread function. Potential formulas were preselected from a theoretical framework as follows: number of carbon = 4–40, hydrogen = 4–80, oxygen = 1–40, nitrogen = 0–1, mass to charge ratio (*m/z*) = 170–700, mass defect (decimal after the nominal mass) = −0.1–0.3, O/C ratio = 0–1, H/C ratio = 0.3–2, double bond equivalence minus oxygen = −10–10 and electron valence must be neutral. These criteria left 11205 potential formulas for assignment to sample peaks.

The data was screened in each transient for instrumental noise by calculating the 95^th^ percentile of peaks with mass defect 0.6–0.8. All intensities occurring below this value at all mass defects were replaced by zero. Formulas were then assigned by comparing each peak in each transient with the exact deprotonated mass of the 11205 formulas in the prepared list. The closest match was assigned if it was within 1.5 ppm of the theoretical deprotonated mass and occurred in at least 10 transients in a sample. 6220 of the formulas were found in at least one sample according to these criteria. The resulting matrix of 6220 formula rows and ~550 transients was then summed into three time bins: A = 5–9.5 min, B = 9.5–19 min, C = 19–24 min. This 6220 × 3 matrix was then normalised to sum 1 × 10^6^. The blanks and three SRFA standards that were analysed at the start, middle and end of the run were assessed and compared for method validation, and not used further. It should be noted that in this separation technique, solvent and buffer composition vary throughout the chromatographic run, making the comparison of signal intensities between fractions problematic. For example, the early fraction has a very low organic solvent content and lower desolvation at the ESI source, leading to lower ionization efficiency of organic compounds in comparison to the later fractions. However, this is not important for inter-sample comparison, as conditions are the same for all samples.

The inter-sample dissimilarity was assessed pairwise using the Bray-Curtis Dissimilarity scale, which was used for classical multidimensional scaling leading to 57 principle coordinates with positive eigenvalues. The analytical reproducibility of the method was high, as three analyses of Suwannee River Fulvic Acid reference material (measured at the start, middle and end of the run) gave a very low Bray-Curtis dissimilarity of 2.23 ± 0.34% (mean ± SD, n = 3). Geochemical and land cover parameters were correlated (Pearson) to PCoA ordinations. Also, the Pearson’s correlation between PCoA scores and each individual formula’s normalised intensity was calculated. Correlations were considered significant if they passed a *p* value of 0.001.

## Electronic supplementary material

Supplementary information

## Data Availability

Raw data and MATLAB processing files are available in the PANGAEA data publisher (https://www.pangaea.de/). https://doi.pangaea.de/10.1594/PANGAEA.895543.
