# Supplementary material for: Regional diversity of complex dissolved organic matter across forested hemiboreal headwater streams
Source: Sci Rep. 2018 Oct 30;8:16060. doi: 10.1038/s41598-018-34272-3 (PMC6207752; doi:10.1038/s41598-018-34272-3)
Supplement: Supplementary file 1 — Supplementary information [file 41598_2018_34272_MOESM1_ESM.docx]

Supplementary information: **Regional diversity of complex dissolved organic matter across forested hemiboreal headwater streams**

Jeffrey A. Hawkes^a,*^, Nikola Radoman^a,¥^, Jonas Bergquist^a^, Marcus Wallin ^b^, Lars Tranvik^c^, Stefan Löfgren^d^

^a^ Analytical Chemistry, Department of Chemistry - BMC, Uppsala University , Uppsala, Sweden
^b^ Department of Earth Sciences, Uppsala University, Uppsala, Sweden
^c^ Department of Limnology, Uppsala University, Uppsala, Sweden
^d^ Department of Aquatic Sciences and Assessment; Section for Geochemistry and Hydrology, Swedish University of Agricultural Sciences (SLU), Uppsala, Sweden
* Corresponding author: [jeffrey.hawkes@kemi.uu.se](mailto:jeffrey.hawkes@kemi.uu.se)
^¥^ Current address: **Department of Environmental Science and Analytical Chemistry, Stockholm University, Stockholm, Sweden**

Contents:

- Extended sampling methodology
- Table SI1: Catchment characteristics in the randomly selected streams compared with at county level in southeast Sweden
- Table SI2: Gradient elution program
- Figure SI1. Gradient program for LC-MS method
- Figure SI2: DOM character statistics
- Table SI2: Correlation values (Pearson’s Rho) between environmental factors or geochemical values and the first three principle coordinates
- Figure SI3: Percentage of total ion current in fraction A vs. sample position on Principle Coordinate 1.
- Figure SI4: Van Krevelen diagram showing direct infusion analysis of an example low TOC sample (SO_033) and high TOC sample (SO_048).
- Figure SI5: Comparison of low and high TOC samples by HPLC-HRMS

Extended sampling methodology

The stream selection methodology applied in this project is based on basic statistical theory, using a randomly selected sub-population for estimating the “true” distribution of different characteristics in the entire population. In short, the headwater streams were identified based on a ‘virtual hydrological network’ generated from a 50 m × 50 m digital elevation model ^1^. The object-oriented database contains water bodies, watersheds and their topological relations. The datasets contains raster data on flow accumulation, flow direction as well as vector data on river reaches, lakes and contributing areas on a national scale. The database includes 930,000 stream reaches/arcs and in this study headwaters are defined as arcs with a start node without discharge from another upstream arc. The threshold value for accumulated flow, defining the initiation of a headwater stream in the landscape, varied between 1.1-3.5 l s^-1^ across Sweden. The water divide for each headwater catchment was modelled using the same elevation data, while land cover data were obtained from classified satellite images (25 m x 25 m pixel, GSD-Marktäckedata, Metria). One hundred headwaters fulfilling the criteria of; being longer than 2,500 m (to ensure that the stream was not ephemeral), without lakes (water surface area ≤1%) and urban areas and with <5% agricultural land in the catchment, were randomly selected. Hence, from a purely statistical point of view, the selected sub-population should reflect the catchment characteristics and stream water chemistry of the hemiboreal streams in southeast Sweden.

This selection methodology generates catchments and streams much smaller than generally used for forest inventories and stream water monitoring. The area of the selected catchments varied in the range 0.95-7.25 km^2^, which is so small that almost no other such data are available. In Table SI1, our headwater catchment and stream data are compared with the same type of data at county level in southeast Sweden. The catchment characteristics and geochemistry ranges are in close agreement, but as expected the randomly selected catchments/streams show larger variation compared with the data aggregated at county level. The chance of having a high proportion of forest land or clear-cuts is larger in a small catchment compared with a large one. Similarly, the chance of having high TOC and low pH is larger in a small stream with high hydraulic connectivity to organic soils compared with surface waters draining large catchments with occurrence of many different soils and potential retention within the aqueous media.

Hence, our conclusion is that the randomly selected sub-population of streams in this study are highly relevant and representative for the hemiboreal region of southeast Sweden. As regards hemiboreal streams outside of this region, it is unknown how representative our study is.

**Table SI1:** Catchment characteristics in the randomly selected streams compared with at county level in southeast Sweden (counties E, F, G, H and K, data from the Swedish National Forest Inventory 2009-2013). Headwater stream geochemistry in the randomly selected streams compared with surface water chemistry data at county level in southeast Sweden (counties E, F, G, H and K, data from the national lake and stream survey 2000 ^2^.

|  | Randomly selected streams -  Range | Randomly selected streams - Median | Southeast Sweden at county level – catchment characteristics | Southeast Sweden at county level – water chemistry |
| --- | --- | --- | --- | --- |
| Catchment area (km^2^) | 0.95-7.25 | 2.34 | Not relevant | Streams: 11.4-222 |
| % Forested area | 48-99 | 83 | 60-78 | No data |
| % Clear-cut area | 1-34 | 14 | 7-18% | No data |
| % Wetland area | 0-44 | 0 | 1-7 | No data |
| TOC (mg/L) | 6.5-63.3 | 27.9 |  | 6.3-33.8 |
| Total Ca (meq/L) | 0.09-3.10 | 0.25 |  | 0.13-1.04 |
| Total Fe (mg/L) | 0.04-3.8 | 0.8 |  | 0.03-1.95 |
| pH | 4.01-7.40 | 4.97 |  | 4.74-7.25 |

**Table SI2:** Chromatographic gradient elution program

| Time | Flow Rate (µL/min) | Solvent B% |
| --- | --- | --- |
| 0.0 | 100 | 0 |
| 1.0 | 100 | 0 |
| 1.1 | 20 | 0 |
| 2.5 | 40 | 0 |
| 3.0 | 40 | 10 |
| 5.0 | 40 | 20 |
| 5.5 | 100 | 20 |
| 7.0 | 100 | 20 |
| 8.0 | 40 | 20 |
| 11.0 | 40 | 20 |
| 12.0 | 40 | 45 |
| 17.0 | 40 | 65 |
| 21.0 | 40 | 90 |
| 22.0 | 100 | 90 |
| 23.0 | 100 | 0 |
| 32.0 | 100 | 0 |

Figure SI1. Gradient program for LC-MS method, with solvent A representing aqueous solution of formic acid and ammonia with pH=3.35 and 5% of acetonitrile, and acetonitrile as solvent B.

The increases in flow rate were used to speed up the chromatography method, so that 74 samples + standards and blanks could be analysed in as short a time frame as possible, maximising MS transient comparability.


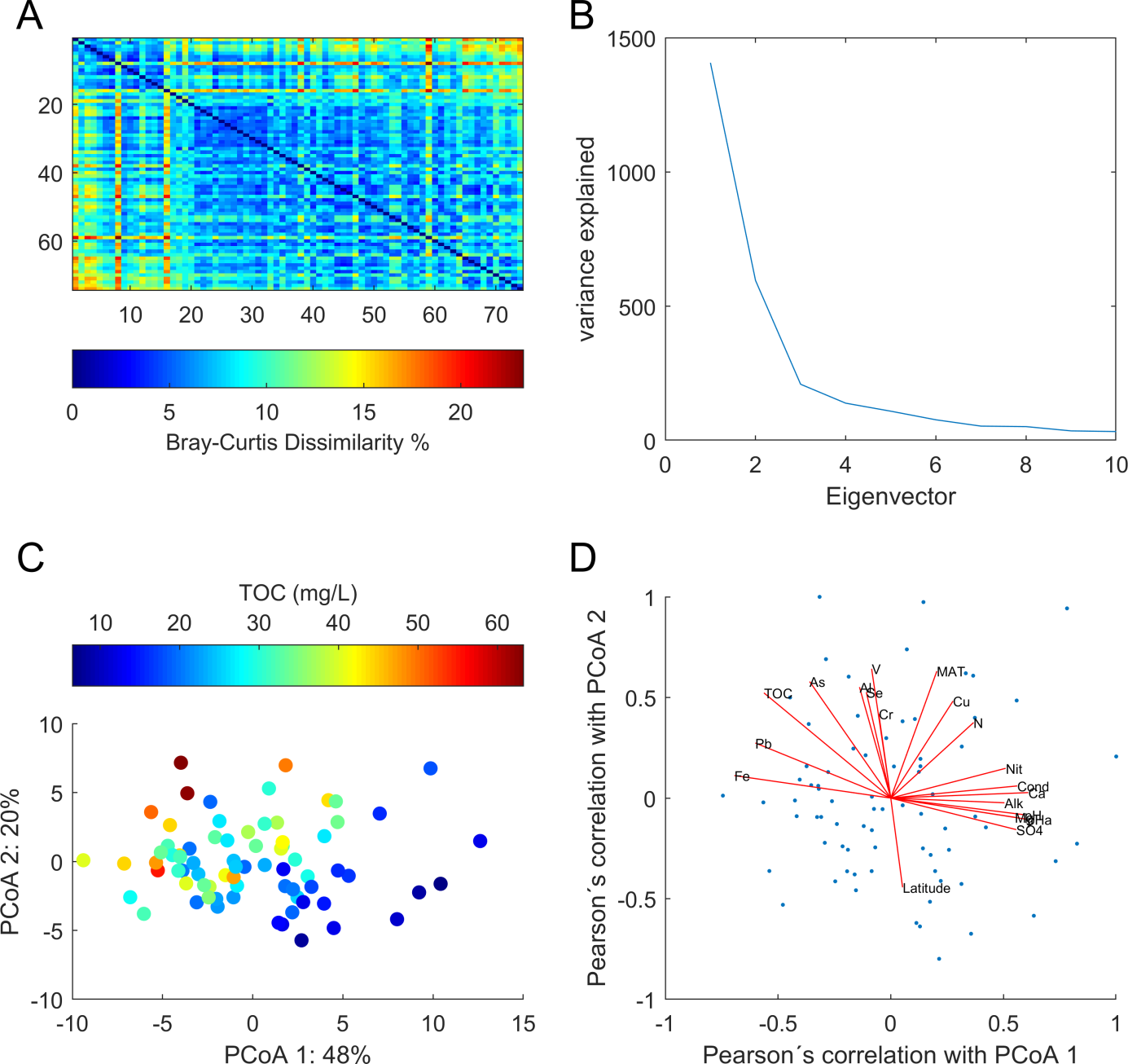


Figure SI2: A: Bray Curtis Dissimilarity matrix with dissimilarity represented by warmth of colour, and samples ordered 1-74 by increasing TOC concentration. B: Scree plot showing variance explained by each principle coordinate after classical multidimensional scaling of the dissimilarity matrix. C: Scores plot showing the 74 sample positions on the first two principle coordinates, with points coloured by TOC. D: Pearson correlation of selected environmental/geochemical data with the principle coordinates 1 and 2. Strength of correlation (Pearson’s Rho) is indicated by the length of the red lines, from 0-1, and is displayed according to each axis. Only factors with correlation significance p<0.001 shown. Latitude negatively correlates with PCoA2, TOC correlates with both axes, negatively with PCoA1 and positively with PCoA2.

Table SI2: Correlation values (Pearson’s Rho) between environmental factors or geochemical values and the first three principle coordinates. Cell colour corresponds to the value and values with p<0.001 are shown in bold.

| Factor | PCoA 1 (47%) | PCoA 2 (21%) | PCoA 3 (7%) |
| --- | --- | --- | --- |
| Temperature during sampling | -0.05 | 0.08 | 0.00 |
| pH | **0.59** | -0.08 | 0.19 |
| pH (aerated sample) | **0.61** | -0.11 | 0.13 |
| Conductivity | **0.56** | 0.06 | -0.16 |
| Alkalinity | **0.50** | -0.02 | 0.28 |
| TOC | **-0.56** | **0.52** | -0.26 |
| Absorbance at 254nm | **-0.57** | **0.53** | -0.16 |
| SUVA254 | -0.22 | 0.33 | 0.30 |
| Total N | **0.37** | 0.38 | -0.10 |
| NH4 | -0.37 | 0.09 | 0.10 |
| Nitrate | **0.51** | 0.15 | -0.06 |
| PO4 | -0.35 | 0.02 | 0.04 |
| Total P | -0.06 | -0.14 | 0.05 |
| Turbidity | -0.37 | 0.06 | -0.16 |
| Suspended Solids | -0.21 | -0.14 | 0.02 |
| SO4 | **0.56** | -0.16 | -0.22 |
| Cl | 0.29 | 0.30 | 0.13 |
| F | 0.27 | 0.20 | 0.19 |
| Ca | **0.61** | 0.03 | -0.09 |
| Mg | **0.55** | -0.10 | -0.23 |
| Na | 0.29 | 0.24 | 0.05 |
| K | 0.22 | 0.06 | 0.19 |
| Fe | **-0.69** | 0.11 | -0.11 |
| Mn | -0.28 | -0.18 | 0.09 |
| Si | 0.22 | 0.22 | -0.14 |
| Al | -0.14 | **0.55** | 0.01 |
| As | -0.36 | **0.58** | -0.17 |
| Cd | -0.23 | 0.01 | 0.00 |
| Co | -0.18 | 0.27 | -0.06 |
| Cr | -0.05 | 0.42 | 0.02 |
| Ni | 0.20 | 0.37 | 0.04 |
| Cu | 0.28 | **0.48** | 0.11 |
| Pb | **-0.60** | 0.27 | -0.12 |
| V | -0.09 | **0.64** | -0.11 |
| Zn | -0.13 | -0.19 | -0.04 |
| Se | -0.11 | **0.53** | 0.11 |
| U | 0.13 | 0.26 | 0.25 |
| Catchment area | 0.12 | 0.07 | 0.10 |
| Clear cut area | 0.21 | -0.14 | -0.05 |
| Agricultural area | 0.15 | -0.10 | 0.04 |
| Forest area | -0.03 | 0.27 | 0.24 |
| Surface water area | -0.24 | -0.02 | 0.13 |
| Wetland area | -0.19 | -0.19 | -0.26 |
| Latitude | 0.05 | **-0.44** | -0.37 |
| Longitude | 0.25 | 0.16 | -0.01 |
| Mean annual temperature (30 year) | 0.20 | **0.63** | 0.17 |
| mean annual precipitation (30 year) | -0.32 | -0.18 | 0.00 |
| Tree volume per hectare | 0.32 | 0.33 | 0.24 |

Figure SI3: Percentage of total ion current in fraction A vs. sample position on Principle Coordinate 1. The samples with higher base cations and lower TOC had a lower percentage of fraction A material.

Figure SI4: Van Krevelen diagrams showing direct infusion analysis of an example low TOC sample (SO_033) and high TOC sample (SO_048). The samples were mixed with acetonitrile and ammonia to final ratios 50:50:0.1 sample:acetonitrile:NH_3_OH, meaning the low TOC sample had lower overall carbon abundance (concentration was not accounted for). The analysis was conducted at the same settings as the LC-MS analysis for the main analysis, so 1 x 10^6^ ions are trapped for each transient, and 150 transients were summed. The point size is proportional to the normalized peak intensity. Two large contaminant peaks with H/C=2 were present in both samples. Many low H/C peaks were not detected in the low TOC sample by direct infusion, whereas they were detected by LC-MS due to the removal of ion suppression in this method.


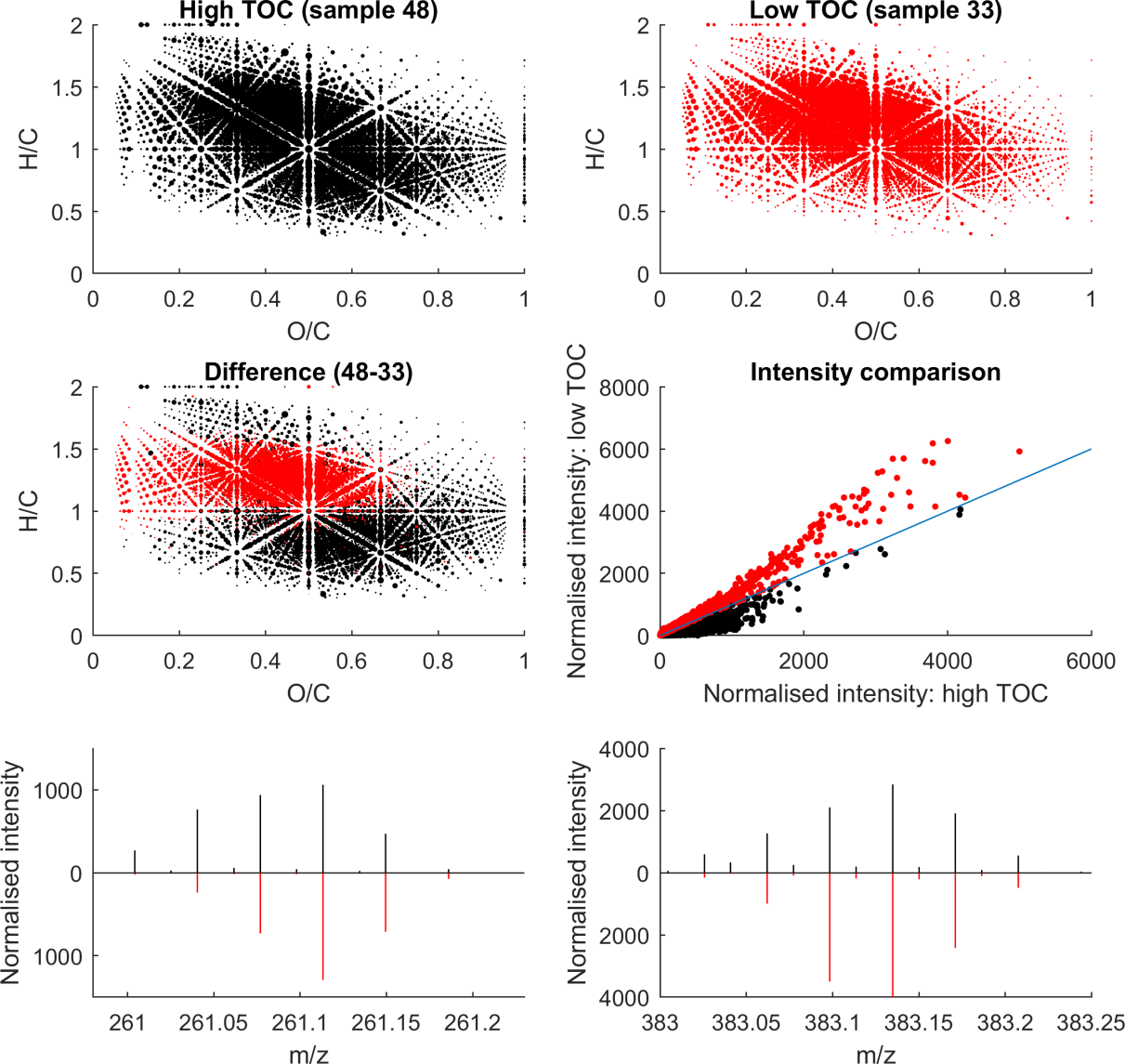


**Figure SI5:** Sample comparison of the two most different samples, based on Bray Curtis dissimilarity: SO_048 (TOC = 40.3 mg L^-1^) and SO_033 (TOC = 12.4 mg L^-1^). The figure shows Van Krevelen diagrams of all three polarity fractions overlaid (top), an intensity comparison (middle) and mass spectrum comparison of two nominal masses (bottom). In the intensity comparison (left), sample SO_033 normalized intensities are subtracted from SO_048 normalized intensities, and resulting negative values are shown in red, with all point sizes scaled to the subtracted intensity value. The peaks that are higher in the high TOC sample are generally lower mass defect peaks (bottom), with lower average intensities (middle right), but this may be due to ionization efficiency at low organic solvent concentration rather than for concentration reasons.

References

1 J. Nisell, A. Lindsjö and J. Temnerud, *Rikstäckande virtuellt vattendrags nätverk för flödesbaserad modellering ViVaN (in Swedish with an English summary). Department of Aquatic Science and Assessment. Swedish University of Agricultural Sciences. Uppsala*, 2007, vol. 17.

2 A. Wilander, R. K. Johnson and W. Goedkoop, *Riksinventeringen 2000 – en synoptisk studie av vattenkemi och bottenfauna i svenska sjöar och vattendrag.*, 2003.
